# *Pbrm1* Loss Induces a Permissive Chromatin State for Cholangiocytic Differentiation and Cholangiocarcinoma Formation

**DOI:** 10.1016/j.jcmgh.2025.101720

**Published:** 2026-01-07

**Authors:** Li-Wen Chiou, Yu-Lin Jhuang, Chia-Lang Hsu, Ray-Hwang Yuan, Yen-Hsu Cheng, Chia-Hsiang Lee, Yi-Ting Fang, Ching-Yao Yang, Yung-Ming Jeng

**Affiliations:** 1 Graduate Institute of Pathology, National Taiwan University, Taipei, Taiwan; 2 Department of Pathology, National Taiwan University Hospital, Taipei, Taiwan; 3 Department of Medical Research, National Taiwan University Hospital, Taipei, Taiwan; 4 Department of Surgery, National Taiwan University Hospital, Taipei, Taiwan; 5 Department of Surgery, National Taiwan University Hospital, Hsinchu Branch, Hsinchu, Taiwan

**Keywords:** Cell Fate, Chromatin Remodeling, Knockout Mice, SNF/SWI Complex

## Abstract

**Background & Aims:**

The SWI/SNF ATP-dependent chromatin remodeling complex regulates transcriptional machinery access and is critical in normal physiology and cancer development. PBRM1, a key subunit of this complex, is frequently mutated in intrahepatic cholangiocarcinoma (iCCA). This study aims to explore the role of PBRM1 in liver physiology and its involvement in iCCA development.

**Methods:**

Liver-specific *Pbrm1* knockout (*Pbrm1* KO) mice were generated to assess the effects of *Pbrm1* loss under various conditions. These mice were exposed to a 3,5-diethoxycarbonyl-1,4-dihydrocollidine diet to induce cholestatic injury and were also subjected to a high-fat diet to evaluate susceptibility to liver steatosis. Chromatin accessibility and gene expression under both normal and injury conditions were examined. Additionally, the impact of *Pbrm1* loss was analyzed in combination with an activating *Kras*^G12D^ mutation to study cancer development.

**Results:**

*Pbrm1* KO mice exhibited increased susceptibility to cholestatic injury, with an enhanced ductular reaction. Loss of *Pbrm1* reduced chromatin accessibility at hepatocyte-specific and metabolically important genes, although RNA expression remained unaffected during homeostasis. Following cholestatic injury, hepatocyte-specific gene expression was significantly reduced compared with wild-type controls. *Pbrm1* KO mice also showed heightened vulnerability to high-fat diet-induced liver steatosis. When combined with *Kras*^G12D^ mutation, *Pbrm1* KO/*Kras*^G12D^ mice had shorter survival and were more likely to develop cholangiocarcinomas, whereas *Pbrm1* wild type/*Kras*^G12D^ mice predominantly developed hepatocellular neoplasms. PBRM1-deficient organoids were highly sensitive to the EZH2 inhibitor tazemetostat, whereas effects on allografts were limited.

**Conclusions:**

PBRM1 maintains chromatin accessibility for hepatocyte differentiation-related genes. Its loss promotes differentiation toward cholangiocytes during injury or tumorigenesis, driving iCCA development.

## Graphical abstract

**Figure:**
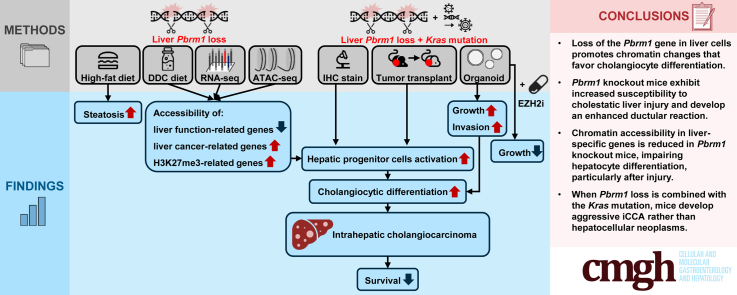

**Summary**

Liver-specific *Pbrm1* deletion reduces chromatin accessibility of hepatocytic genes, enhances cholangiocytic differentiation upon injury, and cooperates with mutant *Kras* to drive intrahepatic cholangiocarcinoma formation, revealing an epigenetic mechanism linking chromatin remodeling to lineage fate and potential EZH2-targeted therapy.

**What You Need to Know**

**Background**

PBRM1, a core subunit of the SWI/SNF chromatin-remodeling complex, is frequently mutated in intrahepatic cholangiocarcinoma. However, how PBRM1 loss alters hepatocyte differentiation and promotes cholangiocarcinogenesis remains poorly defined.

**Impact**

Liver-specific *Pbrm1* deletion decreases chromatin accessibility of hepatocytic genes, enhances cholangiocytic differentiation during injury, and cooperates with mutant *Kras* to drive intrahepatic cholangiocarcinoma formation.

**Future Directions**

Further studies should investigate how other chromatin remodelers compensate for Pbrm1 loss and whether these compensatory mechanisms can be exploited as therapeutic targets.

Intrahepatic cholangiocarcinoma (iCCA) is the second most common primary liver cancer after hepatocellular carcinoma (HCC). The incidence of iCCA varies greatly across the globe, with higher prevalence in East and South Eastern Asia compared with Western regions.^1^ This elevated rate in Asia is primarily attributed to infections by liver flukes, specifically *Clonorchis sinensis* and *Opisthorchis viverrini*.^2^^,^^3^ Other contributing factors to iCCA include hepatolithiasis, primary sclerosing cholangitis, exposure to the radiopaque medium thorium dioxide (Thorotrast), biliary tract abnormalities, and infections with hepatitis B and C viruses.^4^^,^^5^

Extensive molecular profiling of iCCA has uncovered a wide range of genomic alterations.^6^, ^7^, ^8^, ^9^, ^10^ *KRAS* mutations are found in 10% to 20% of iCCAs, whereas mutations in isocitrate dehydrogenase 1 (*IDH1*) and 2 (*IDH2*) occur in about 10% of cases.^6^, ^7^, ^8^ Additionally, gene fusions involving *FGFR2* present actionable therapeutic targets.^9^ Exome sequencing has frequently identified inactivating mutations in chromatin-remodeling genes such as *BAP1*, *ARID1A*, and *PBRM1* in iCCA.^10^ Notably, mutations in *IDH1/2*, *BAP1*, and *PBRM1*, as well as *FGFR2* fusions, are rarely observed in other hepatobiliary cancers like hilar cholangiocarcinoma, extrahepatic cholangiocarcinoma, and HCC, suggesting that iCCA has a distinct origin and pathogenesis.

The SWItch/Sucrose NonFermentable (SWI/SNF) chromatin remodeling complexes regulate chromatin accessibility for transcriptional and regulatory processes.^11^ Inactivating mutations in subunits of the SWI/SNF complexes are commonly identified in various cancers, highlighting their tumor-suppressive roles. Two main SWI/SNF complexes exist: the BRG1/BRM-associated factor (BAF) and polybromo-associated BAF (PBAF).^12^ PBRM1, also known as BAF180, is a key subunit of the PBAF complex, which features 2 bromo-adjacent homology domains that mediate protein interactions and 6 bromodomains that bind to histone H3 at specific acetyl-lysine sites.^13^ PBRM1’s primary function is to regulate the assembly of the PBAF complex and its recruitment to specific genomic loci to modulate gene expression.^11^

Loss-of-function mutations in *PBRM1* are common in multiple cancers, most notably clear-cell renal cell carcinoma, iCCA, and chordoma, suggesting a tumor-suppressive role for PBRM1.^10^^,^^14^, ^15^, ^16^ Despite its frequent mutation in iCCA, the specific roles of PBRM1 in iCCA pathogenesis, as well as in maintaining hepatocyte and cholangiocyte homeostasis, remain unclear. In this study, genetically engineered mouse models were used to explore the functions of PBRM1 in liver biology and its contribution to iCCA tumorigenesis.

## Results

### Loss of Pbrm1 Promotes the Activation of Hepatic Progenitor Cells in Response to Cholestatic Injury

**Figure 1:**
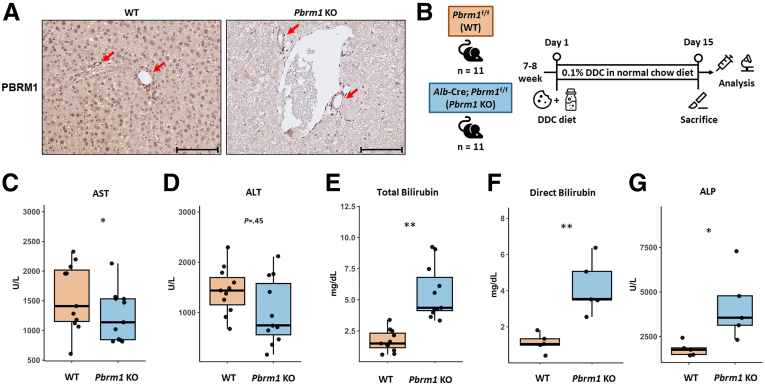
**Loss of *Pbrm1* enhances the activation of hepatic progenitor cells in response to cholestatic injury.** (*A*) Immunohistochemical staining confirms the loss of PBRM1 expression in hepatocytes and cholangiocytes in the interlobular bile ducts of *Pbrm1* KO mice. Scale bar: 100 μm. (*B*) Overview of the DDC diet treatment protocol. (*C*–*G*) Serum levels of AST (*C*), ALT (*D*), total bilirubin (*E*), direct bilirubin (*F*), and ALP (*G*) in WT and *Pbrm1* KO mice following DDC diet treatment (n = 11 for each group for *A*–*E*, n = 5 for each group for *F* and *G*). ∗*P* < .05 and ∗∗*P* < .01.

To investigate the role of PBRM1 in liver function and tumorigenesis, we crossed mice with a loxP-flanked *Pbrm1* allele with *Alb*-Cre mice, generating *Alb*-Cre; *Pbrm1*^fl/fl^ mice (referred to as *Pbrm1* knockout [KO] mice). Immunostaining of *Pbrm1* KO livers showed loss of nuclear PBRM1 expression in hepatocytes and cholangiocytes in the interlobular bile ducts, whereas it remained in endothelial, stromal, inflammatory cells, and cholangiocytes in the larger bile ducts (Figure 1*A*). There were no significant differences in survival rates, body weights, or liver appearance between *Pbrm1* KO and wild-type (WT) mice over 12 months.

**Figure 2:**
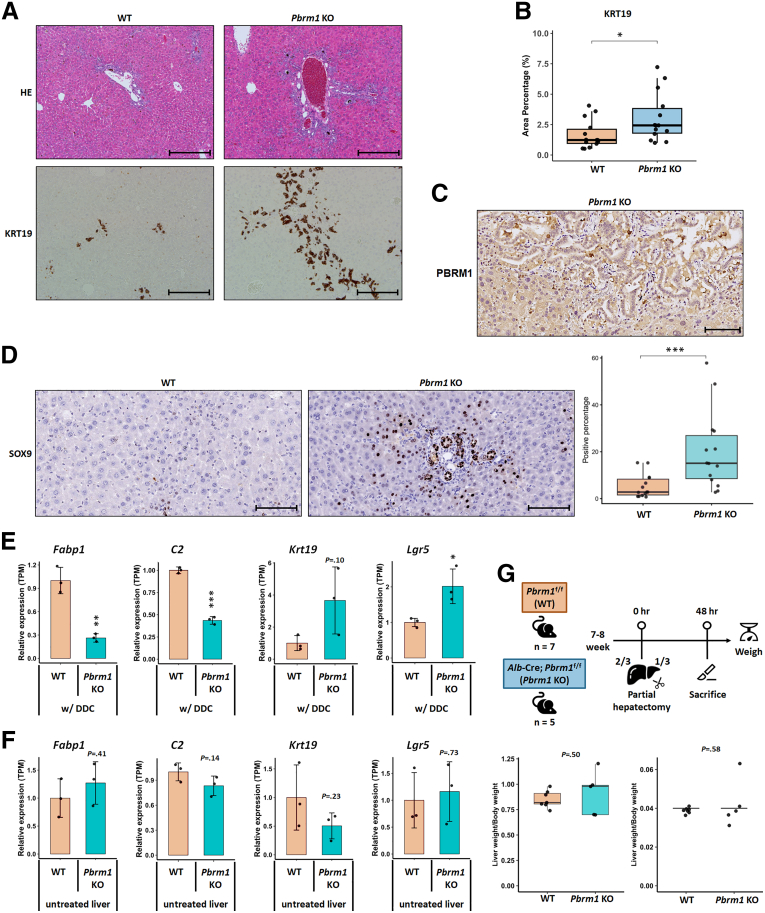
**Histologic and molecular evidence of enhanced hepatic progenitor cell activation in response to cholestatic injury in *Pbrm1* KO liver.** (*A*) Histological analysis of H&E and KRT19-stained sections reveals increased bile ductular proliferation in the livers of *Pbrm1* KO mice after DDC diet treatment. (*B*) Quantification of KRT19-positive areas in the livers of WT and *Pbrm1* KO mice post-DDC diet treatment (n = 14 for each group). (*C*) Immunostaining demonstrates loss of PBRM1 expression in exaggerated ductular reaction in *Pbrm1* KO liver. (*D*) Quantification of SOX9-positive hepatocytes in the livers of WT and *Pbrm1 KO* mice post-DDC diet treatment (n = 14 for each group). (*E* and *F*) RNA-seq data shows downregulation of hepatocyte markers *Fabp1* and *C2,* and upregulation of cholangiocyte marker *Krt19* and progenitor marker *Lgr5* in *Pbrm1* KO mice on the DDC diet (*E*), with no differences observed in untreated mice (n = 3 for each group) (*F*). (*G*) Forty-eight hours post-partial hepatectomy, the liver-to-body weight ratio, an indicator of liver regeneration, shows no significant difference between *Pbrm1* KO (n = 5) and WT mice (n = 7). ∗*P* < .05; ∗∗*P* < .01; and ∗∗∗*P* < .001. Scale bars for (*A*): 500 μm; for (*C*) and (*D*): 100 μm.

In response to injury, liver regeneration typically involves the proliferation of mature hepatocytes or expansion of hepatic progenitor cells (HPCs).^17^ Although hepatocyte proliferation drives regeneration after partial hepatectomy or acute injury, chronic or cholestatic injury activates HPCs, leading to differentiation into hepatocytes and cholangiocytes, causing a ductular reaction. Mice aged 7 to 8 weeks were fed a 0.1% 3,5-diethoxycarbonyl-1,4-dihydrocollidine (DDC)-supplemented diet for 2 weeks (Figure 1*B*). Although aspartate aminotransferase (AST) and alanine aminotransferase (ALT) levels were similar between *Pbrm1* KO and WT mice (Figure 1*C* and *D*), *Pbrm1* KO mice showed elevated total bilirubin, direct bilirubin, and alkaline phosphatase (ALP), indicating more severe cholestatic injury (Figure 1*E–G*). Histology revealed greater porphyrin plug accumulation and a more pronounced ductular reaction, marked by KRT19, in *Pbrm1* KO mice (Figure 2*A* and *B*). The ductal cells exhibited loss of PBRM1 expression, indicating that the exaggerated ductular reaction is intrinsic to this cell population rather than the response to hepatocyte injury (Figure 2*C*). Previous studies have shown that SOX9^+^ hepatocytes function as bipotent progenitors after liver injury, contributing to both hepatocyte and ductal cell lineages during regeneration^18^^,^^19^ In the present study, immunostaining revealed a marked expansion of Sox9^+^ hepatocytes in *Pbrm1* KO livers compared with WT livers after DDC diet treatment, implying that *Pbrm1* loss may promote hepatocyte-to-cholangiocyte transdifferentiation (Figure 2*D*). RNA sequencing (RNA-seq) data confirmed downregulation of hepatocyte markers *Fabp1* and *C2*, and upregulation of cholangiocyte marker *Krt19* and progenitor marker *Lgr5* in *Pbrm1* KO mice on the DDC diet, with no differences observed in untreated mice (Figure 2*E* and *F*).

Further analysis of liver regeneration after partial hepatectomy showed no difference in liver weight recovery between *Pbrm1* KO and WT mice (Figure 2*G*). These findings suggest that *Pbrm1* loss enhances liver regeneration through hepatic progenitor cell expansion but does not affect regeneration via mature hepatocyte proliferation.

### Pbrm1 Loss Decreases Chromatin Accessibility of Genes Related to Hepatocytic Differentiation

**Figure 3:**
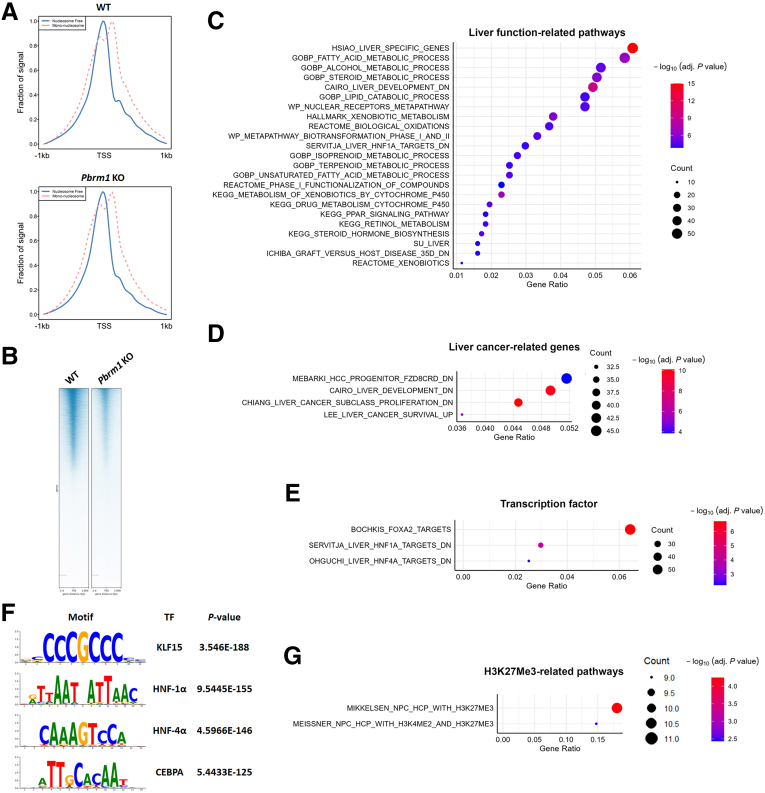
**Loss of *Pbrm1* reduces chromatin accessibility of genes associated with hepatocyte differentiation.** (*A*) ATAC-seq peaks are concentrated near TSSs in both WT and *Pbrm1* KO livers. (*B*) Overall decrease in chromatin accessibility observed in *Pbrm1* KO livers. (*C*) ATAC-seq reveals reduced chromatin accessibility in liver-specific genes, particularly those related to hepatocyte differentiation and metabolism, in *Pbrm1* KO livers. (*D*) ATAC-seq shows decreased chromatin accessibility in genes downregulated during tumor progression in *Pbrm1* KO livers. (*E*) ATAC-seq demonstrates reduced accessibility in genes regulated by hepatocyte-enriched transcription factors such as HNF-4α, HNF-1α, and FOXA2 in *Pbrm1* KO livers. (*F*) Motif analysis indicates diminished accessibility at binding sites for liver-enriched transcription factors KLF15, HNF-1α, HNF-4α, and CEBPA in *Pbrm1* KO livers. (*G*) Two gene sets linked with H3K27me3 show increased accessibility in *Pbrm1* KO livers. (n = 3 for each group).

Given PBRM1’s role as a chromatin remodeler, we examined chromatin accessibility changes using Assay for Transposase-Accessible Chromatin Using Sequencing (ATAC-seq). As expected, ATAC peaks were concentrated near transcription start sites (TSSs) (Figure 3*A*). In *Pbrm1* KO mice, we observed a general reduction in chromatin accessibility, indicating that PBRM1 mainly facilitates chromatin opening to allow transcription factor access (Figure 3*B*). Of 1365 significantly different ATAC peaks (adjusted *P* value < .05), 80 sites had increased accessibility, whereas 1285 showed reduced accessibility in *Pbrm1* KO livers. Each peak was mapped to the nearest gene. Pathway analysis revealed that the genes with reduced accessibility in *Pbrm1* KO livers were liver-specific genes, particularly those involved in hepatocyte differentiation and metabolism, such as xenobiotic, fatty acid, steroid, and alcohol metabolism pathways (Figure 3*C*).

Furthermore, genes downregulated in the ‘proliferation’ subclass of HCC and genes associated with better survival in HCC also had decreased chromatin accessibility in *Pbrm1* KO mice (Figure 3*D*). Lineage-specific transcription factors guide differentiation, and genes regulated by hepatocyte-enriched transcription factors like hepatocyte nuclear factor (HNF)-4α, HNF-1α, and FOXA2 exhibited reduced chromatin accessibility in *Pbrm1* KO livers (Figure 3*E*). Motif analysis confirmed decreased accessibility at binding sites for liver-enriched transcription factors, including KLF15, HNF-1α, HNF-4α, and CEBPA (Figure 3*F*). Conversely, only 2 gene sets associated with histone H3K27 trimethylation (H3K27me3) showed increased accessibility in *Pbrm1* KO livers (Figure 3*G*).

### Correlation of Chromatin Accessibility and RNA Expression in Mice With or Without DDC Diet Treatment

**Figure 4:**
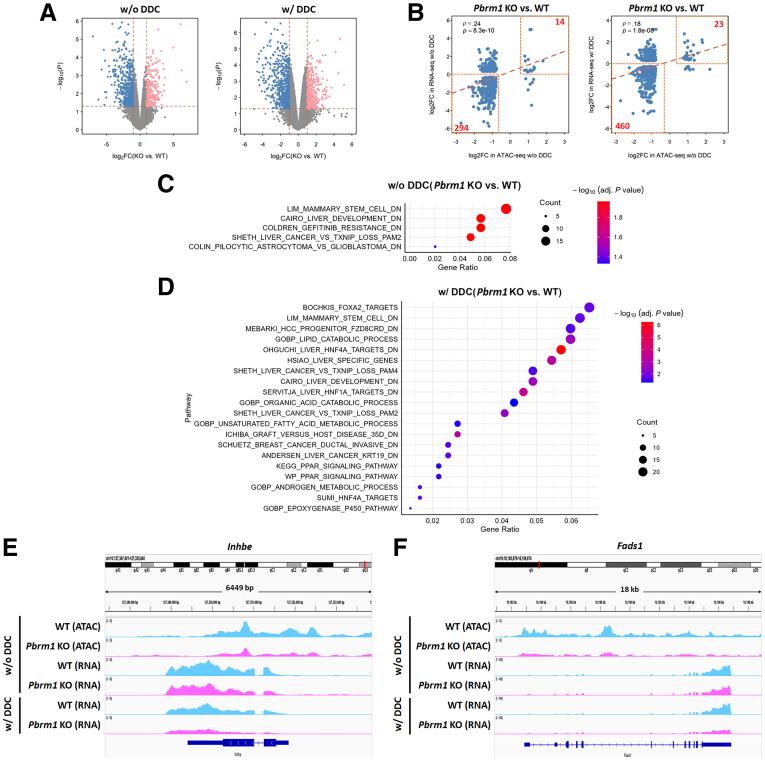
**Correlation between chromatin accessibility and RNA expression in mice with or without DDC diet treatment.** (*A*) Volcano plots display differentially expressed genes between *Pbrm1* KO and WT livers, both with and without DDC diet treatment. *Pbrm1* KO livers exhibit a greater number of downregulated genes compared with WT in both conditions. (*B*) Correlation analysis of RNA expression and chromatin accessibility in *Pbrm1* KO and WT livers with or without DDC diet treatment. (*C* and *D*) Pathway analysis of genes that are downregulated and exhibit reduced chromatin accessibility in *Pbrm1* KO livers without (*C*) and with (*D*) DDC diet treatment. (*E* and *F*) *Inhbe* (*E*) and *Fads1* (*F*) show reduced chromatin accessibility in *Pbrm1* KO livers without DDC diet treatment, whereas RNA expression remains similar between *Pbrm1* KO and WT. However, after DDC diet treatment, RNA expression of these genes decreases in *Pbrm1* KO mice. (n = 3 for each group).

RNA-seq was performed on 3 biological replicates of liver tissues from both WT and *Pbrm1* KO mice, with and without DDC diet treatment. In *Pbrm1* KO mice without DDC diet treatment, we identified 213 upregulated genes and 510 downregulated genes, using a threshold of fold change ≥2 and *P* < .05. After DDC diet treatment, 233 genes were upregulated, and 463 were downregulated (Figure 4*A*). Integrating RNA-seq with ATAC-seq data revealed concordant changes in gene expression and DNA accessibility near promoters or enhancers in both conditions (Figure 4*B*). However, in untreated *Pbrm1* KO livers, the 294 genes showing concordant changes in transcription and chromatin accessibility were not associated with liver differentiation or function pathways, consistent with the absence of morphological abnormalities in *Pbrm1* KO livers (Figure 4*C*).

In contrast, after DDC diet treatment, the genes with reduced expression and chromatin accessibility were enriched for liver-specific functions, including metabolic processes and targets of HNF-4α and HNF-1α (Figure 4*D*). For instance, genes like *Fads1*, involved in fatty acid metabolism, and *Inhbe*, a hepatocyte-specific secretory protein, exhibited reduced chromatin accessibility in *Pbrm1* KO livers. Although RNA expression of these genes was similar in untreated livers, their expression significantly declined upon DDC diet treatment (Figure 4*E* and *F*). Therefore, while *Pbrm1* loss does not immediately affect liver morphology and function, it shifts chromatin remodeling towards reduced hepatocellular differentiation. Upon cholestatic injury, *Pbrm1* loss biases the regenerative response toward cholangiocytic differentiation.

### Pbrm1 Loss Increases Susceptibility to High-fat Diet-induced Fatty Liver

**Figure 5:**
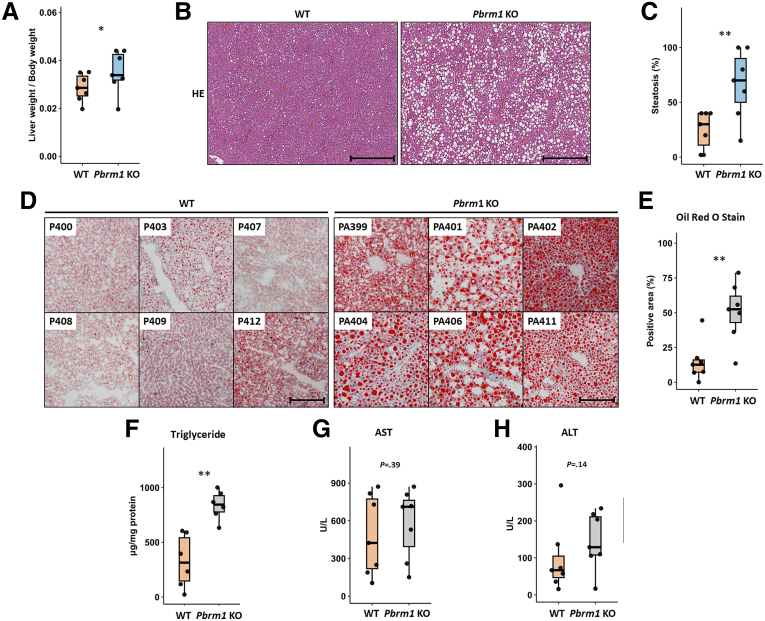
**Loss of *Pbrm1* increases vulnerability to high-fat diet-induced fatty liver.** (*A*) After high-fat diet treatment, the liver weight/body weight ratio is increased in *Pbrm1* KO mice (n = 7 for each group). (*B* and *C*) Following high-fat diet treatment, *Pbrm1* KO livers exhibit more pronounced fatty changes in H&E-stained histological sections (n = 7 for each group). Scale bar: 500 μm. (*D* and *E*) Fresh frozen liver specimens stained with Oil Red show increased fat droplet accumulation in *Pbrm1* KO liver, with quantification of fat-occupied areas (n = 7 for each group). Scale bar: 500 μm. (*F*) Quantification of triglyceride levels in *Pbrm1* KO and WT livers using a colorimetric assay (n = 6 for each group). (*G* and *H*) Serum levels of AST (*G*) and ALT (*H*) are similar in both *Pbrm1* KO and WT mice after high-fat diet treatment (n = 7 for each group). ∗*P* < .05 and ∗∗*P* < .01.

A key function of the liver is regulating lipid metabolism. Disruption of hepatic lipid balance leads to fatty liver disease, a global health issue affecting up to 20% of the population.^20^ Molecular pathway analysis showed underexpression of several fat metabolism pathways in *Pbrm1* KO mice (Figure 4*D*). To investigate the role of PBRM1 in lipid homeostasis, *Pbrm1* KO and WT mice were fed a high-fat diet for 13 weeks. The liver weight/body weight ratio was increased *Pbrm1* KO mice (Figure 5*A*). Histological analysis revealed more intracytoplasmic fat vacuoles in *Pbrm1* KO livers (Figure 5*B* and *C*). Oil Red O staining confirmed an increased area of oil droplets in *Pbrm1* KO liver tissue, indicating greater steatosis (Figure 5*D* and *E*), which was further supported by higher triglyceride levels (Figure 5*F*). However, steatosis in *Pbrm1* KO mice was not associated with inflammatory cell infiltration or hepatocyte death. Serum levels of AST and ALT were similar in *Pbrm1* KO and WT mice (Figure 5*G* and *H*), indicating that the high-fat diet in this model led to simple steatosis without progression to steatohepatitis.

### Cooperation of Pbrm1 Loss and Mutant Kras in iCCA Tumorigenesis

**Figure 6:**
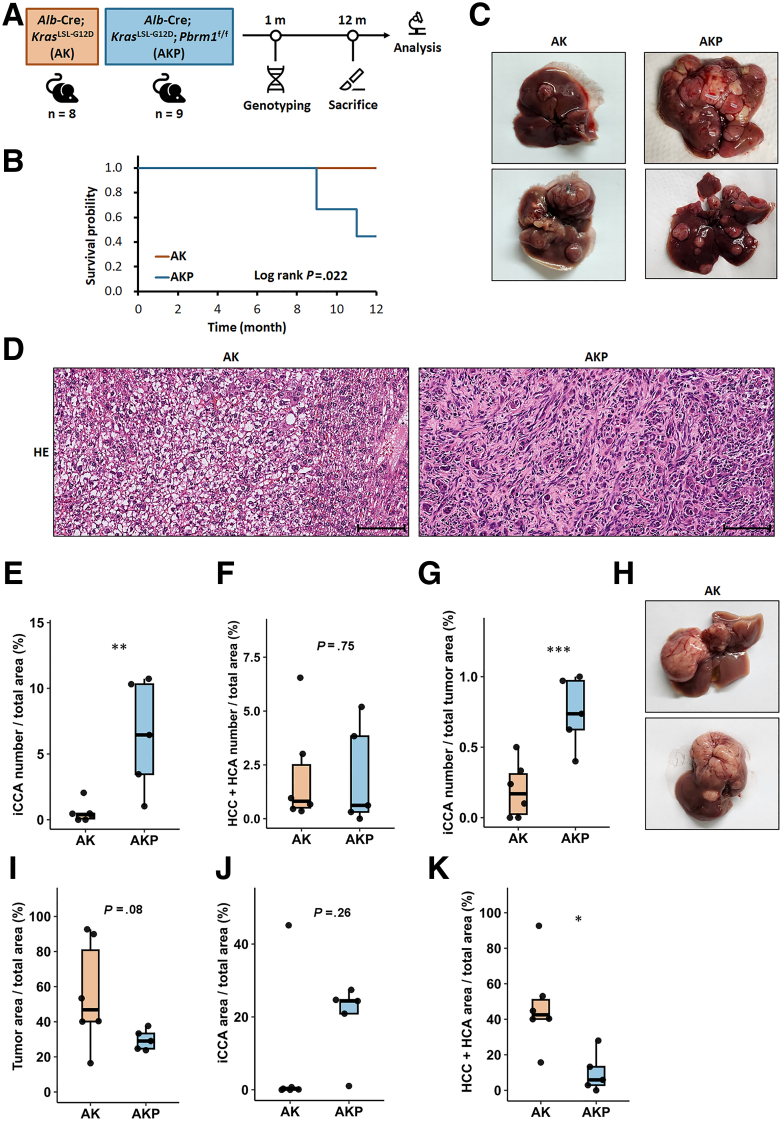
**Interaction between *Pbrm1* loss and mutant *KRAS* in iCCA development.** (*A*) Schematic overview of the mouse tumorigenesis model. (*B*) Kaplan-Meier analysis comparing AK (n = 8) and AKP male mice (n = 9) showing the time until illness requiring euthanasia or up to 12 months. (*C*) Representative images of AK and AKP livers, both displaying multiple tumors. (*D*) Histological images showing HCC in an AK mouse and iCCA in an AKP mouse. Scale bar: 100 μm. (*E*–*G*) Age-matched AKP livers have a higher density of iCCAs per unit area (*E*), whereas the number of hepatocellular neoplasms (HCA and HCC) is similar between AK and AKP livers (*F*). The percentage of iCCAs in the total tumor count is significantly higher in AKP livers (*G*). (*H*) Photographs of 2 AK livers with a dominant HCC in each liver. (*I*) The total tumor areas per unit area is larger in AK livers. (*J*) The total iCCA tumor areas per unit area is larger in AKP livers. (*K*) The total hepatocellular neoplasm tumor areas per unit area is larger in AK livers. (n = 6 for AK mice and n = 5 for AKP mice in *E*–*K*). ∗*P* < .05; ∗∗*P* < .01; and ∗∗∗*P* < .001.

**Figure 7:**
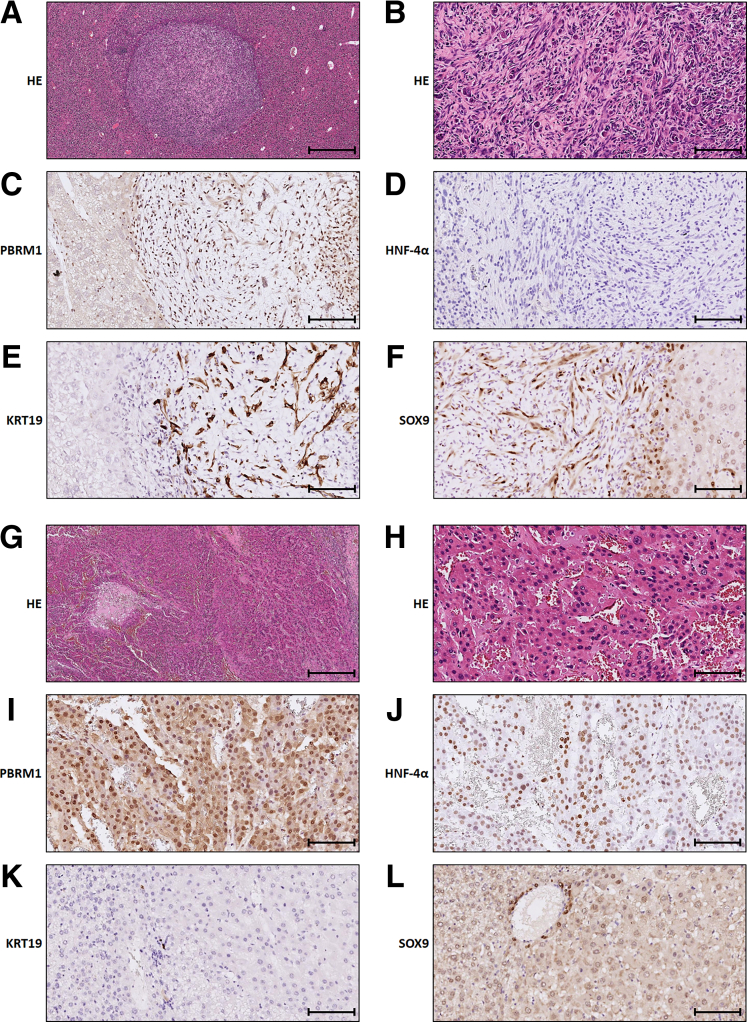
**Representative histology and immunophenotype of tumors from AKP and AK mice.** (*A*–*F*) Histology and immunophenotype of an iCCA derived from an AKP liver. Low magnification (*A*, 40×) and high magnification (*B*, 200×) views of an H&E-stained section show tumor cells arranged in glandular patterns or as isolated cells within a fibrous stroma. IHC analysis reveals that the tumor cells do not express PBRM1 (*C*) or HNF-4α (*D*), but are positive for KRT19 (*E*) and SOX9 (*F*). Histology and immunophenotype analysis of an HCC derived from an AK liver. (*G* and *H*) Low magnification (*G*, 40×) and high magnification (*H*, 200×) views of an H&E-stained section reveal tumor cells with abundant eosinophilic cytoplasm arranged in trabecular patterns. IHC staining shows that the tumor cells express PBRM1 (*I*) and HNF-4α (*J*), but lack expression of KRT19 (*K*) and SOX9 (*L*). Scale bars for (*A*) and (*G*): 500 μm; *B*-*F* and *H*-*L*: 100 μm.

To investigate the role of PBRM1 in liver tumor development, we generated 2 Kras-driven mouse models by crossing *Alb*-Cre mice with *Kras*^LSL-G12D^ and *Pbrm1*^f/f^ mice, resulting in *Alb*-Cre; *Kras*^LSL-G12D^ mice (AK mice) and *Alb*-Cre; Kras^LSL-G12D^; *Pbrm1* ^f/f^ mice (AKP mice) (Figure 6*A*). Due to notable sex differences in liver tumor models,^21^^,^^22^ only male mice were used in this study. Five of 9 AKP mice died between 9 and 11 months, whereas all AK mice survived up to 12 months (Figure 6*B*). At the study’s endpoint, both AK and AKP mice developed multiple liver tumor nodules (Figure 6*C*). Histological analysis revealed the presence of hepatocellular neoplasms (hepatocellular adenoma [HCA] or HCC) and iCCA in both groups (Figure 6*D*). iCCAs displayed tumor cells arranged in glandular patterns or as single cells within the fibrous stroma, expressing cholangiocyte markers KRT19 and SOX9, and lacking hepatocyte marker HNF-4α (Figure 7*A*–*F*). HCA and HCC were composed of hepatocyte-like cells arranged in trabecular patterns, confirmed by positive staining for hepatocyte marker HNF-4α and negative for cholangiocyte markers KRT19 and SOX9 (Figure 7*G*–*L*). AKP mice had significantly more iCCAs compared with AK mice, although both genotypes exhibited similar numbers of hepatocellular neoplasms (Figure 6*E* and *F*). Consequently, the proportion of iCCAs in AKP mice was significantly higher than in AK mice (Figure 6*G*).

Due to the frequent occurrence of dominant HCCs in AK mice (Figure 6*H*), the liver areas occupied by tumors are greater in AK mice (Figure 6*I*). Among these, iCCA tumor areas are larger in AKP mice, whereas hepatocellular neoplasm areas are larger in AK mice (Figure 6*J* and *K*).

**Figure 8:**
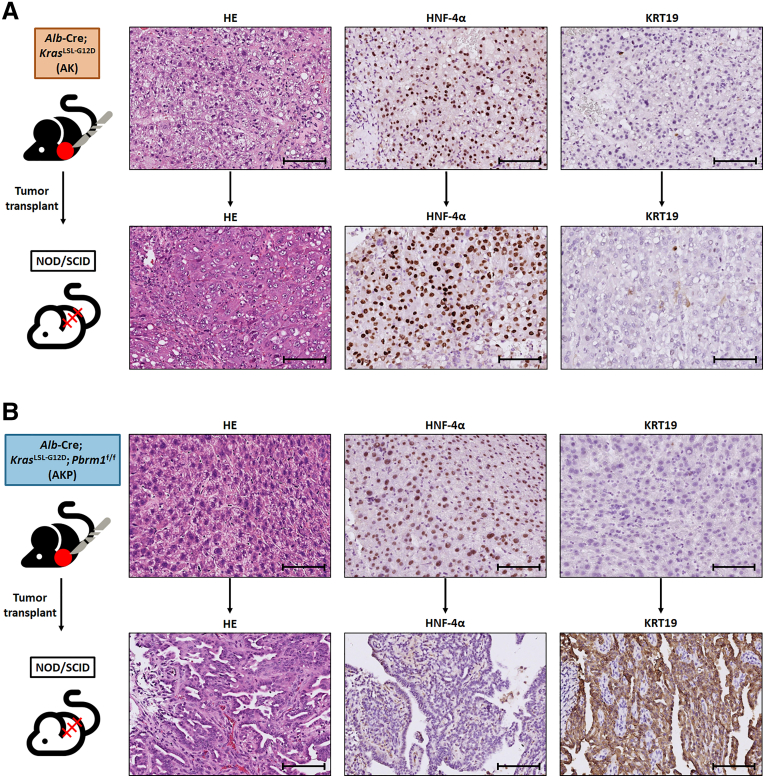
**Tumors from one HCC in an AK mouse and one HCC in an AKP mouse were transplanted into the subcutaneous tissue of NOD/SCID mice.** (*A*) The tumor from the AK mouse maintained its HCC morphology, showing HNF-4α expression, but lacked KRT19 expression. (*B*) In contrast, the tumor from the AKP mouse progressed into iCCA, with a loss of HNF-4α expression and gained expression of KRT19. Scale bar: 100 μm.

We attempted to generate tumor allografts by transplanting liver tumors into the subcutis of non-obese diabetic/severe combined immunodeficient (NOD/SCID) mice. However, 3 iCCAs were unable to form tumors in the NOD/SCID mice over 6 months. One HCC from AK mice and 2 HCC from AKP mice were successfully transplanted into NOD/SCID mice. Interestingly, the tumor from the AK mouse retained the morphology of HCC, whereas the tumors from the AKP mouse transformed into iCCA (Figure 8). These phenotypes were maintained after retransplantation.

Thus, aside from increasing the frequency of tumor initiation, a key effect of *Pbrm1* loss is promoting differentiation toward cholangiocytes during tumorigenesis.

### Liver Organoid Model Shows That Pbrm1 Loss Promotes Cell Growth and Invasion in a Cell-autonomous Manner

**Figure 9:**
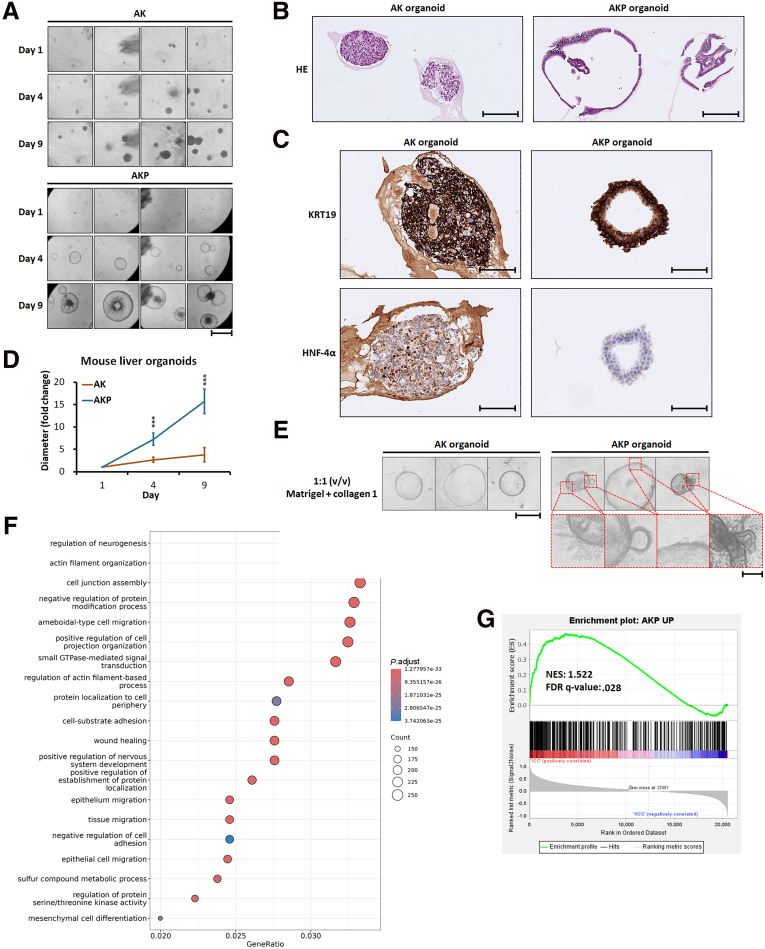
**Cholangiocytic differentiation and enhanced proliferation and invasiveness of AKP liver organoids.** (*A*) The phase-contrast images of AK and AKP organoids. Scale bar: 1 mm. (*B*) The H&E-stained sections of AK and AKP organoids. Scale bar: 100 μm. (*C*) Immunostaining shows both AK and AKP organoids expressed KRT19. HNF-4α is expressed in AK organoids and not expressed in AKP organoids. Scale bar: 100 μm. (*D*) AKP liver organoids (n = 9) exhibit a significantly higher growth rate compared with AK organoids (n = 14). (*E*) AKP organoids exhibits more protruding spikes compared with AK organoids when cultured in Matrigel supplemented with collagen 1. Scale bar for the left panel: 500 μm; for the right panel:100 μm. (*F*) GSEA demonstrates the enriched Gene Ontology pathways in AKP organoids. (*G*) GSEA of RNA-seq data of 372 HCC and 36 iCCA samples in TCGA PanCancer Atlas shows that genes predominantly expressed in AKP organoids were significantly enriched in those highly expressed in iCCA. ∗∗∗*P* < .001.

Our mouse model mirrors human iCCA in featuring a desmoplastic stroma, which has been proven to play diverse roles in influencing tumor cell differentiation, growth, and invasion.^23^^,^^24^ Thus, one potential mechanism through which PBRM1 contributes to liver tumorigenesis could involve interactions between tumor cells and the microenvironment. To determine if PBRM1 drives tumorigenesis in a cell-autonomous manner, we generated liver organoids from AK and AKP mice. As shown in Figure 9*A* and *B*, liver organoids from AK mice displayed a solid sphere structure, whereas those from AKP mice formed lumen-containing spheres, resembling previously described hepatocyte and biliary epithelial cell organoids.^25^ Although both AK and AKP organoids expressed KRT19, HNF-4α expression was entirely absent in AKP organoids, indicating a loss of hepatocytic differentiation (Figure 9*C*). Additionally, the loss of *Pbrm1* significantly promoted organoid growth (Figure 9*D*). Our previous research demonstrated that the interaction between liver cancer cells and collagen 1 enhances tumor invasion.^24^ Therefore, we cultured liver organoids in Matrigel supplemented with collagen 1. AKP organoids exhibited more protruding spikes compared with AK organoids, indicating that *Pbrm1* loss enhances cell invasiveness (Figure 9*E*).

To profile how *Pbrm1* loss affects liver organoids, we performed RNA-seq to compare the transcriptomes of AK and AKP organoids. As expected, given the cholangiocytic differentiation of AKP organoids, they exhibited high expression of cholangiocytic markers *Krt19* and *Krt7*. Consistent with the enhanced cell invasiveness of AKP organoids, pathway analysis revealed that AKP organoids were enriched in pathways associated with tumor invasion, including actin filament organization, amoeboid-type cell migration, positive regulation of cell projection organization, and regulation of actin-filament-based processes (Figure 9*F*). To study the relevance to human liver tumors, we used the genes upregulated in AKP organoids to generate a gene expression signature, which we then applied to publicly available RNA-seq data from 372 HCC and 36 iCCA samples in the Cancer Genome Atlas (TCGA) PanCancer Atlas. Gene set enrichment analysis (GSEA) demonstrated that genes predominantly expressed in AKP organoids were significantly enriched in those highly expressed in iCCA (Figure 9*G*).

### Pbrm1 Loss Increases Sensitivity to EZH2 Inhibitors

**Figure 10:**
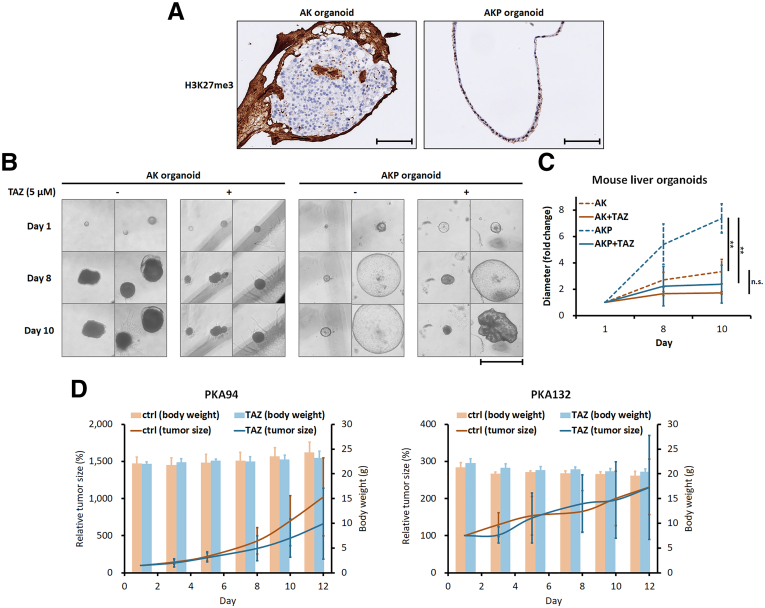
**The expression levels of H3K27Me3 in liver organoids and their responsiveness to the EZH2 inhibitor TAZ.** (*A*) Immunostaining demonstrates higher H3K27Me3 levels in AKP organoids. Scale bar: 100 μm. (*B* and *C*) TAZ treatment reduces the elevated growth of AKP liver organoids (n = 4 for each group, 3 biological replicates). Scale bar: 1 mm. (*D*) Tumor cells were injected subcutaneously into the flanks of NOD-SCID mice. When tumors were established (mean tumor size: 150 mm^3^), the tumors were randomized into 2 groups (n = 10 per group) and treated with vehicle or TAZ (200 mg/kg) by oral gavage daily. The tumor sizes and mouse body weights were measured every 2 to 3 days. ∗∗*P* < .01, n. s.: not significant.

Previous studies have shown that loss-of-function mutations in SWI/SNF complex subunits, including *PBRM1*, lead to increased activity of enhancer of zeste homolog 2 (EZH2), a subunit of the Polycomb repressive complex 2 (PRC2) responsible for trimethylating histone 3 at lysine 27.^26^ EZH2 inhibitors have been shown to induce synthetic lethality and apoptosis in other *PBRM1*-mutated cancer models.^27^, ^28^, ^29^ The only 2 gene sets with increased chromatin accessibility in *Pbrm1* KO livers were related to the H3K27me3 chromatin marker (Figure 3*G*). Immunostaining revealed that AKP organoids exhibited higher levels of H3K27me3 compared with AK organoids (Figure 10*A*). Thus, we tested the effect of the EZH2 inhibitor tazemetostat (TAZ) on liver organoids and found that the enhanced growth of AKP liver organoids was significantly inhibited by TAZ treatment (Figure 10*B* and *C*). To evaluate the in vivo effects of EZH2 inhibition on tumor growth, we derived 2 cancer cell lines from AKP mouse HCCs and implanted them into the flanks of NOD/SCID mice. Animals received either vehicle or TAZ (200 mg/kg) for 12 days. In one cell line, TAZ modestly reduced tumor size, although the difference did not reach statistical significance. The second cell line showed no response to treatment (Figure 10*D*). Body weight remained stable across all groups, indicating minimal toxicity at the administered dose.

## Discussion

In this study, we introduce new genetically engineered mouse models to examine the role of PBRM1 in liver homeostasis and the development of iCCA. *Pbrm1* knockout did not alter liver histology or function, indicating that PBRM1 is not essential for normal liver development and function. Additionally, the absence of tumor formation in *Pbrm1* KO mice suggests that *Pbrm1* loss alone is insufficient for tumorigenesis. We further investigated the role of PBRM1 in liver injury response. Following hepatotoxic injury, the liver regenerates by increasing hepatocyte mitosis and differentiating stem cells into hepatocytes or cholangiocytes.^30^ Liver regeneration mechanisms depend on the injury type and severity: after partial hepatectomy, remaining hepatocytes proliferate to restore liver mass, whereas severe or cholestatic injury enables biliary epithelial cells to dedifferentiate into progenitor cells with potential to differentiate into hepatocytes and bile duct cells.^30^ The DDC diet model, which induces cholestatic liver injury via intraductal porphyrin plug formation, showed that *Pbrm1* loss enhances the ductular reaction, indicating increased activation of liver progenitor cells. However, liver regeneration after partial hepatectomy was unaffected by *Pbrm1* loss. Thus, *Pbrm1* loss specifically heightens progenitor-driven regeneration without impacting hepatocyte-based liver regeneration.

Because PBRM1 is part of the PBAF chromatin remodeling complex, we used ATAC-seq to examine the effects of *Pbrm1* knockout on chromatin accessibility in liver cells, finding a general decrease in accessibility, particularly in liver-specific genes and those linked to hepatocyte function. Additionally, we observed reduced accessibility in genes downregulated in the ‘proliferation’ subclass of HCC, aligning with clinical observations that *PBRM1* loss-of-function mutations are common in iCCA but not HCC. To further investigate whether changes in chromatin accessibility affected gene expression, we performed RNA-seq, identifying a positive correlation between PBRM1-regulated open chromatin regions and gene expression in untreated mice. This correlation was even stronger with DDC diet treatment, indicating that PBRM1 maintains open chromatin states for hepatocyte-specific genes during homeostasis, whereas its loss reduces chromatin accessibility and promotes differentiation towards cholangiocytes following injury. The permissive chromatin state is critical for regulating cell fate during development, especially in multipotent progenitor cells.^31^^,^^32^ Similar mechanisms have been reported with Ari1a, another SNF/SWI complex subunit that maintains open chromatin in hepatocytes to support their response to liver injury.^33^

Although *Pbrm1* encodes a core component of the PBAF chromatin-remodeling complex, its loss alone did not cause obvious abnormalities in liver development or hepatocyte differentiation under physiological conditions. This observation is consistent with previous KO studies of other SWI/SNF subunits, including *Brg1* (*Smarca4*), *Brd7,* and *Baf60a* (*Smarcd1*), whose deletion in hepatocytes similarly results in morphologically and functionally normal livers.^34^, ^35^, ^36^ In contrast, loss of *Arid1a* and *Arid2*, which are key regulatory subunits of the canonical and PBAF complexes, respectively, leads to spontaneous hepatic steatosis—appearing at approximately 3 months in *Arid1a*-deficient and 7 months in *Arid2*-deficient mice—highlighting subunit-specific functions within the SWI/SNF family.^37^^,^^38^ The lack of a developmental phenotype after *Pbrm1* loss likely reflects the redundant or compensatory activities of other chromatin remodelers that preserve essential transcriptional programs in the quiescent liver. Moreover, chromatin accessibility alone does not guarantee transcriptional activation; gene expression requires the coordinated action of transcription factors, enhancers, and co-activators, and a merely permissive chromatin landscape may be insufficient to drive lineage-specific or stress-induced gene expression in the absence of appropriate regulatory cues. Together, these considerations suggest that PBRM1 primarily modulates transcriptional responses under pathological or stress conditions, rather than during normal liver homeostasis.

Mutations in driver genes often regulate both cell proliferation and differentiation. For instance, *IDH1/2* gain-of-function mutations, frequently found in iCCA but not HCC, inhibit hepatocyte differentiation by repressing HNF-4α, a key regulator of hepatocyte identity.^39^ Additionally, mutant *IDH* and *KRAS* cooperate to expand liver progenitor cells, promoting biliary intraepithelial lesion formation and iCCA progression.^39^ Expression of *FGFR2* fusions in mouse liver organoids similarly drives tumor formation towards cholangiocarcinoma,^40^ and hepatocyte-specific *KRAS*^G12D^ expression combined with *BAP1* loss leads to rapid iCCA development.^41^ Our findings suggest that *Pbrm1* loss represents another mechanism hindering hepatocyte differentiation, contributing to iCCA development. *Pbrm1* loss has also been shown to block proximal tubule differentiation in kidney cancer^42^ and prevent progenitor-to-pre-myelinating cell transition during oligodendrocyte maturation.^43^

Tumor-specific mutations may present opportunities for targeted therapy. In *Droso*phila, antagonistic interactions between Polycomb group genes and the SWI/SNF complex have been identified.^44^ EZH2, the catalytic subunit of PRC2, catalyzes H3K27 trimethylation.^45^ Kim et al demonstrated that EZH2 is essential for cancer cell survival in cancer cell lines and xenografts with mutations of the SWI/SNF subunits *ARID1A*, *PBRM1*, and *SMARCA4*.^27^ EZH2 inhibitor TAZ had dramatic in vivo efficiency in *PBRM1*-mutated human chordoma xenograft.^28^ Our ATAC-seq results show that only 2 pathways with increased chromatin accessibility in *Pbrm1* KO liver are those related to H3K27me3 chromatin marks. We therefore tested the EZH2 inhibitor TAZ, which is approved for metastatic epithelioid sarcoma treatment,^45^ in our organoid model. TAZ significantly inhibited growth in AKP liver organoids. However, TAZ produced only a modest growth-inhibitory effect in one of the AKP cell lines. The limited response is likely multifactorial. Unlike liver organoids, cancer cell lines often harbor additional genetic or epigenetic alterations that may blunt the impact of EZH2 inhibition. Moreover, the allografts derived from these cultured lines consist largely of undifferentiated tumor cells, which may be less responsive to the differentiation-inducing effects of TAZ observed in primary tumors. Technical factors—such as dosing or treatment schedule—may also have influenced the outcome. Notably, a recent report described a patient with *PBRM1*-mutated metastatic cholangiocarcinoma who achieved a durable partial response to TAZ, remaining on therapy for over 30 months without dose reduction or interruption.^46^ This encouraging clinical example indicates that TAZ may be a promising therapeutic option for patients with *PBRM1*-mutated iCCA; however, larger, well-designed studies are needed to establish the clinical benefits of EZH2 inhibition in this setting.

In summary, although PBRM1 is not necessary for liver development or function under homeostatic conditions, it maintains chromatin accessibility for genes associated with hepatocyte differentiation. Its loss facilitates differentiation towards cholangiocytes in response to injury or during tumorigenesis, promoting iCCA progression. Our findings also support clinical exploration of EZH2 inhibitors for treating iCCAs with *PBRM1* mutations.

## Materials and Methods

### Animal Experiments

Animal studies were conducted following guidelines of the Council of Agriculture, Taiwan, and approved by the Institutional Animal Care and Use Committee of Medical School, National Taiwan University (approval number 20210252). Mice had access to food ad libitum unless otherwise specified and were housed in a temperature- and light-controlled, specific pathogen-free facility. The mice were genotyped using standard polymerase chain reaction (PCR) protocols, with primers listed in Table 1. Both male and female mice with the appropriate genotype were included in the experiments unless otherwise specified. The mice were randomly assigned into each group in studies of cholestatic injury, hepatectomy, and high-fat diet treatment. At the end of the study, the mice were sacrificed after euthanized via CO_2_ suffocation.

**Table 1:** The Primers Used for Genotyping

	Primer ID	Sequence 5' → 3'	Primer type
Pbrm1	31922	GAC ATG GCT TCT CCC AAA CT	Forward
	31923	TGC AAC TCT TTG TCC TTA CAC G	Reverse
Alb-cre	20239	TGC AAA CAT CAC ATG CAC AC	WT forward
	20240	TTG GCC CCT TAC CAT AAC TG	Common
	oIMR5374	GAA GCA GAA GCT TAG GAA GAT GG	Mutant forward
Kras	LSL Kras mutant	AGC TAG CCA CCA TGG CTT GAG TAA GTC TGC	Forward
	LSL Kras WT	CCT TTA CAA GCG CAC GCA GAC TGT AGA	Reverse

WT, wild-type.

The *Pbrm1*^f/f^ strain (B6;129-*Pbrm1*^tm1Zhwa/J^), *Kras*^LSL-G12D^ strain (B6.129S4-*Kras*^tm4Tyj/J^), and *Alb*-Cre strain (B6.Cg-Speer6-ps1^Tg(*Alb*-cre)21Mgn/J^) were obtained from the Jackson Laboratories. To generate the desired genotypes, *Pbrm1*^f/f^ mice were initially crossed with *Alb*-Cre, producing *Alb*-Cre; *Pbrm1*^f/+^ offspring. These mice were then crossed with *Pbrm1*^f/f^ mice to obtain *Alb*-Cre; *Pbrm1*^f/f^ mice (*Pbrm1* KO). Additionally, *Pbrm1*^f/f^ mice were crossed with *Kras*^LSL-G12D^ mice to create *Kras*^LSL-G12D^; *Pbrm1*^f/+^ mice, which were then crossed with *Pbrm1*^f/f^ mice to generate *Kras*^LSL-G12D^; *Pbrm1*^f/f^ mice. These mice were further crossed with *Alb*-Cre; *Pbrm1*^f/f^ mice to produce *Alb*-Cre; *Kras*^LSL-G12D^; *Pbrm1*^f/f^ mice (AKP mice). Finally, *Alb*-Cre mice were crossed with *Kras*^LSL-G12D^ mice to produce *Alb*-Cre; *Kras*^LSL-G12D^ mice (AK mice).

### Mouse Model of Cholestatic Injury

Seven- to 8-week-old mice were fed a diet supplemented with 0.1% DDC (Sigma-Aldrich, Cat.#137030) for 2 weeks to induce cholestatic damage. Blood samples were collected via cardiac puncture post-sacrifice, centrifuged at 3000 rpm for 20 minutes, and serum was isolated. Levels of serum AST, ALT, bilirubin, and ALP were measured by the Laboratory Animal Core at National Taiwan University Medical School and the Taiwan Mouse Clinic at Academia Sinica. Liver tissue was fixed in 10% formalin and processed for paraffin embedding.

### Two-thirds Partial Hepatectomy

Two-thirds hepatectomy was performed following the method described by Claudia Mitchell and Holger Willenbring.^47^ Mice were sacrificed 48 hours post-surgery. The liver-to-body weight ratio was measured to evaluate liver regeneration.

### High-fat Diet Treatment

Six- to 7-week-old mice were housed in a barrier facility with free access to water and a high-fat diet with 58 kcal % from fat and sucrose (Research Diet D12331i, Research Diets) to induce hepatosteatosis. After 13 weeks on the high-fat diet, the mice were sacrificed for further analysis.

### Histology and Immunohistochemical Staining

Tissue samples were fixed in neutral-buffered formalin and embedded in paraffin. Hematoxylin and eosin (H&E)-stained slides were reviewed by a pathologist (Y.M.J.) for histological examination. For Oil Red O staining, frozen liver tissue sections (10 μm) were air-dried for 30 minutes, fixed in neutral-buffered formalin for 10 minutes, placed in propylene glycol for 2 minutes, and stained with 0.5% Oil Red O in propylene glycol at 37°C for 6 minutes. After staining, slides were washed with distilled water, transferred to 50% isopropanol, and counterstained with hematoxylin.

Organoid samples were scraped from the cell culture plate and immersed in ice-cold Advanced Dulbecco’s Modified Eagle Medium (DMEM)/F-12 medium (Gibco, Cat.#11320033) to dissolve the Matrigel (Corning, Cat.#356237). After fixation in neutral-buffered formalin, fixed organoids were embedded in 2% agarose. The solidified mixture of organoids and agarose was enclosed in a cassette and subjected to the following immunohistochemical (IHC) staining process in a similar procedure to that of tissue samples.

For IHC staining, 5-μm sections were dewaxed, rehydrated, and underwent antigen retrieval by incubation in 0.01 M citric acid buffer at 100°C for 10 minutes. After blocking with 3% hydrogen peroxide and 5% fetal bovine serum (FBS), the slides were incubated with primary antibodies overnight at 4°C. When mouse monoclonal antibodies were used, a mouse-on-mouse blocking reagent (Mouse on Mouse Polymer IHC Kit, Abcam, Cat. #ab269452) was applied before the primary antibody incubation. The following day, the slides were treated with a polymer-horseradish peroxidase (HRP) reagent (N-Histofine Simple Stain Mouse MAX PO, Cosmo Bio USA, Cat. # NIC-414351F), and peroxidase activity was visualized using a diamino-benzidine tetrahydrochloride solution (Roche, Cat.#253-4582). Sections were counterstained with hematoxylin. Antibody details and dilutions are listed in Table 2.

**Table 2:** The Antibodies Used for Immunohistochemical Staining

Target	Dilution	Catalog number	Vendor
KRT19	1:100	ab133496	Abcam
HNF4A	1:100	ab199431	Abcam
PBRM1	1:100	A301-591A	Bethyl
SOX9	1:100	HPA001758	Sigma-Aldrich
H3K27me3	1:100	ab6002	Abcam

All microscopic images were captured from whole-slide scans using a Hamamatsu NanoZoomer S360 slide scanner (Hamamatsu Photonics).

### ATAC-seq

After euthanasia, liver tissues were snap-frozen and used for nuclei isolation as previously described.^48^ Nuclei isolated from 50,000 cells underwent transposition using a Nextera DNA Library Prep Kit (Illumina). The resulting DNA was column-purified and amplified in 50-μL reactions using NEBNext High-Fidelity 2× PCR Master Mix (New England Biolabs) and barcoded primers (Genomics). The prepared library was sequenced on an Illumina NovaSeq 6000 platform to generate 150-bp paired-end reads.

### ATAC-seq Data Analysis

ATAC-seq reads were trimmed using Cutadapt (v3.5). The trimmed reads were then aligned to the mouse genome (mm39) with BWA-mem (v0.7.17). For downstream analysis, only reads on autosomes, excluding ENCODE human blacklisted regions and PCR duplicates identified by GATK-Picard (v4.1.9.0), were retained. Aligned reads were adjusted by shifting +4 and −5 bp for positive and negative strands using deepTools’ alignmentSieve (v3.5.0). The genomic distribution of these reads relative to TSSs was examined using deepTools. Peaks were called with MACS2 (v2.2.7.1) using parameters -f BAMPE, -keep-dup all, and -q 0.05. Peaks from individual samples were merged with bedtools (v2.29.2), and merged peaks were annotated using the ChIPseeker R package. Footprinting and transcription factor binding were analyzed using TOBIAS with JASPAR (8th release) motifs. Bedtools was used to calculate read counts for each ATAC peak, which were normalized to counts per million for further analysis. Differential ATAC peaks were identified using the limma R package. Over-representation analysis of genes located near the differentially accessible ATAC peaks (adjusted *P* value < .05) was performed using the clusterProfiler R package with gene sets from MSigDB (v7.4).

### RNA-seq and Analysis

Total RNA was extracted from liver tissue using Trizol reagent (Thermo Fisher Scientific, Cat. #15596018). For mRNA sequencing, RNA quality and quantity were evaluated using a Bioanalyzer 2100 (Agilent) and a Qubit 2.0 Fluorometer (Thermo Fisher Scientific). RNA was ligated to adapters for further amplification using a TruSeq Stranded mRNA Library Prep kit (Illumina). The obtained library was sequenced on an Illumina NovaSeq 6000 sequencer. Post-sequencing quality control was done using FastQC. The adaptor sequences were trimmed using Cutadapt (v3.5). The trimmed reads were aligned to the mouse reference genome (mm39) using STAR (v 2.7.8a) with 2-pass mode, and then gene counts were generated (--quantMode GeneCounts) based on the annotation of Gencode (v35). Normalization was performed using the Trimmed Mean of M values method to ensure comparable distributions across samples, and transcript per million (TPM) values were calculated for subsequent analyses. Differential expression analysis was performed using the limma R package. Over-representation analysis of genes with differentially accessible ATAC peaks and differential expression was performed using the clusterProfiler R package with gene sets from MSigDB (v7.4).

### Organoid Culture

AK and AKP mice were euthanized with CO_2_ and dissected within 2 hours post-mortem. The entire liver was collected to isolate hepatic progenitor cells. Hepatic progenitor organoids were established and maintained following the HepaticCult Organoid Growth Medium protocol (StemCell Technologies, Cat. #06030). For investigating invasion ability, organoids were cultured in Matrigel supplemented with collagen 1 (Corning, Cat. #354231) for the ratio of 1:1 (v/v). For treatments with the EZH2 inhibitor, TAZ (MedChemExpress, Cat. #HY-13803) was dissolved in dimethyl sulfoxide (DMSO). Both the medium and the inhibitor were refreshed every 3 days.

### Xenograft Experiment

A total of 5 × 10^6^ tumor cells were subcutaneously implanted into the flanks of 6-week-old female NOD/SCID mice. Mice were treated with vehicle or TAZ (200 mg/kg) by oral gavage daily for 12 days. Mouse body weights and tumor sizes were monitored every 2 to 3 days. Tumor volume was calculated using the formula: 1/2 × (length in mm × (width in mm)^2^). At the end of the experiment, mice were sacrificed, and tumors were collected and weighed. Investigators were not blinded to the treatment groups during the experiment.

### Sex as a Biological Variable

All animal experiments for tumorigenesis in this study were performed using male mice. No human participants or sex-specific human materials were included. Therefore, the results apply to male mice only.

### Statistical Analysis

The results were analyzed using GraphPad Prism 9.5.1 or R (version 4.3.2; R Foundation for Statistical Computing). Data were first tested for normality using Shapiro’s test. For a comparison of 2 groups, a 2-tailed unpaired Student’s *t*-test was used. For a comparison of multiple groups, the statistical analyses included 1-way or 2-way analysis of variance (ANOVA), followed by post-hoc Tukey testing of pairwise comparisons. Significance was established at *P* < .05.

## Declaration of Generative AI and AI-assisted Technologies in the Writing Process

During the preparation of this work, the authors used chatGPT in order to improve readability and language. After using this tool/service, the authors reviewed and edited the content as needed and take full responsibility for the content of the publication.
